# Probiotic fermentation improves the bioactivities and bioaccessibility of polyphenols in *Dendrobium officinale* under *in vitro* simulated gastrointestinal digestion and fecal fermentation

**DOI:** 10.3389/fnut.2022.1005912

**Published:** 2022-09-07

**Authors:** Rurui Li, Zhenxing Wang, Kin Weng Kong, Ping Xiang, Xiahong He, Xuechun Zhang

**Affiliations:** ^1^Key Laboratory for Forest Resources Conservation and Utilization in the Southwest Mountains of China, Ministry of Education, Southwest Forestry University, Kunming, China; ^2^College of Life Science, Southwest Forestry University, Kunming, China; ^3^Department of Molecular Medicine, Faculty of Medicine, University of Malaya, Kuala Lumpur, Malaysia; ^4^Institute of Environmental Remediation and Human Health, Southwest Forestry University, Kunming, China; ^5^College of Horticulture and Landscape, Southwest Forestry University, Kunming, China

**Keywords:** fermentation, *Dendrobium officinale*, bioaccessibility, *in vitro* digestion, fecal fermentation, gut microbiome, short-chain fatty acid

## Abstract

The objective of the research was to investigate and compare the bioactivities and bioaccessibility of the polyphenols (PPs) from *Dendrobium officinale* (DO) and probiotic fermented *Dendrobium officinale* (FDO), by using *in vitro* simulated digestion model under oral, gastric and intestinal phases as well as colonic fermentation. The results indicated that FDO possessed significantly higher total phenolic contents (TPC) and total flavonoid contents (TFC) than DO, and they were released most in the intestinal digestion phase with 6.96 ± 0.99 mg GAE/g DE and 10.70 ± 1.31 mg RE/g DE, respectively. Using high-performance liquid chromatography (HPLC), a total of six phenolic acids and four flavonoids were detected. In the intestinal phase, syringaldehyde and ferulic acid were major released by DO, whereas they were p-hydroxybenzoic acid, vanillic acid, and syringic acid for FDO. However, apigenin and scutellarin were sustained throughout the digestion whether DO or FDO. As the digestive process progressed, their antioxidant ability, α-amylase and α-glucosidase inhibitory activities were increased, and FDO was overall substantially stronger in these activities than that of DO. Both DO and FDO could reduce pH values in the colonic fermentation system, and enhance the contents of short-chain fatty acids, but there were no significantly different between them. The results of the 16S rRNA gene sequence analysis showed that both DO and FDO could alter intestinal microbial diversity during *in vitro* colonic fermentation. In particular, after colonic fermentation for 24 h, FDO could significantly improve the ratio of Firmicutes to Bacteroidetes, and enrich the abundancy of *Enterococcus* and *Bifidobacterium* (*p* < 0.05), which was most likely through the carbohydrate metabolism signal pathway. Taken together, the PPs from DO and FDO had good potential for antioxidant and modulation of gut bacterial flora during the digestive processes, and FDO had better bioactivities and bioaccessibility. This study could provide scientific data and novel insights for *Dendrobium officinale* to be developed as functional foods.

## Introduction

*Dendrobium officinale* Kimura et Migo (*Dendrobium officinale*, DO), which belongs to the family Orchidaceae, is a perennial epiphytic herbal plant and has been widely used in many Asian countries because of its high nutritional and medicinal value (1, 2). As a well-known homologous medicine and food worldwide, DO has been reported to contain abundant active ingredients such as alkaloids, polysaccharides, phenanthrenes, anthrones, sesquiterpenoids, polyphenols, etc., and demonstrate many biological activities including antioxidant, antitumor, hypoglycemic, and immunomodulatory activities (3). However, most of the studies focused on the common bioactive component in DO such as polysaccharides and alkaloids, whereas minimal studies were carried on its polyphenols (PPs), another kind of important secondary metabolites (4–6). Previous studies reported the abundant PPs in DO, such as rutin, quercitrin, hyperoside, and quercetin, that were classified as flavonoids. In addition, phenolic acids and their derivatives including syringic acid, dihydro ferulic acid tyramine, p-hydroxybenzoic acid, ellagic acid, and gallotannins were also discovered in DO. These PPs were shown to have various biological activities, such as antioxidant, hypoglycemic, anticancer, antimicrobial, and immunomodulatory activities (7, 8).

However, large numbers of polyphenol-rich foods, including DO, are still underutilized due to their low bioavailability. The bioavailability of PPs depends on their bioaccessibility, which is defined as the fraction of these phenolic compounds released in the gastro-intestinal tract, not only just the mere phenolic content in the food. Many factors such as food matrix, solubility, digestibility, molecular structures, or metabolizing enzymes can greatly influence bioaccessibility (9). For example, PPs complexation with fiber, starch, pectin, proteins may significantly influence the bioaccessibility and activity of PPs (10). Therefore, fermentation appears to be an effective method to strengthen the physiological activities of PPs in DO (11). Microbial-mediated fermentation can degrade the macromolecules in food which later promote the release of active substance, to be better absorbed and utilized by the body (12, 13). It has been shown that the biological activities of PPs can be promoted through microbial fermentation, such as anti-inflammatory and antioxidative stress effects, while the effects of fermentation on the bioaccessibility of PPs in DO during gastrointestinal digestion were still unknown (14).

In general, human digestion is a multistep process that can be divided into four major stages: oral phase, gastric phase, small intestine phase, and colonic fermentation phase. During the oral process, food could be fragmented after chewing and digested by digestive enzymes, especially carbohydrates. Then the oral content flows, including protein, fiber, and fat, through the esophagus into the stomach and is digested by gastric acid and enzymes. After digestion in the stomach for several hours, the contents enter the small intestine and large intestine where it is retained and undergoes digestion and absorption (15, 16). During the intestinal phase, the gut microbiota promotes digestion and food absorption for host energy production, whereas in the colon, complex carbohydrates are fermented and converted by the microbiota into short chain fatty acids (SCFAs), a kind of microbial metabolites beneficial to host immune system and energy metabolism, such as n-butyrate, acetate, and propionate (17).

There is growing evidence that the gut microbiota plays a key role in modulating the production, bioavailability, and biological activities of PPs (18), and the inter-individual differences in the composition of gut microbiota have different effects (19). Conversely, PPs also can modulate the colonic microbial population composition or activity (20, 21). Therefore, it is highly necessary to investigate the efficacy in the absorption through the gastrointestinal tract of PPs and the interrelation between PPs and gut microbiota. Although *in vivo* digestions best represent the digestion of food in real conditions, *in vitro* digestion models and continuous colonic culture fermentation models are widely utilized owing to their advantages such as low price, speed, less labor-intensive, and no restrictions on humanities and morality (22). Nevertheless, to date, there is a limited report on the digestion stability of PPs from DO and FDO, and their bioaccessibility at *in vitro* digestion traits.

In this study, we fermented DO with a probiotic (*Lactobacillus delbrueckii* subspecies *bulgaricus*), then investigated the release of bioactive compounds, antioxidant activities, and bioaccessibility of PPs from DO and fermented DO (FDO) under gastrointestinal digestion and colonic fermentation. In addition, the influence of DO and FDO on human fecal microbiota and SCFAs were also evaluated. We expect that our results could provide new insights into the application and further development of DO.

## Materials and methods

### Chemicals and reagents

The *Dendrobium officinale* (DO) under the forest for 3–4-year-old was collected in Wenshan County, Kunming City, Yunnan Province, China, in June 2021.

Chemicals and reagents such as potassium chloride (KCl), dipotassium hydrogen phosphate (K_2_HPO_4_), magnesium chloride hexahydrate (MgCl_2_6H_2_O), sodium chloride (NaCl), hydrochloric acid (HCl), and ammonium carbonate (NH_4_)_2_CO_3_, 6-hydroxy-2,5,7,8-tetramethylchroman-2-carboxylic acid (Trolox), 2,2′-azino-bis-(3-ethylbenzothiazoline-6-sulfonic acid) (ABTS), 2,2-diphenyl-1-picrylhydrazyl (DPPH), 2,4,6-Tris(2-pyridyl)-s-triazine (TPTZ), vitamin C (Vc), and 2,6-di-tert-butyl-4-methylphenol (BHT) were purchased from Sinopharm Chemical Reagent (Beijing, China). Acetonitrile (chromatographic grade) was purchased from Merck (Darmstadt, Germany). Other reference standards were supplied by Yuanye Bio-Technology (Shanghai, China). The pancreatin (from porcine gastric mucosa), and pepsin (from porcine gastric mucosa) were from Ruijin Medical Devices Co. Ltd (Jiangxi, China). The α-glucosidase (from *Saccharomyces* cerevisiae), α-amylase (from hog pancreas), acarbose, P-nitrophenyl-α-D-glucopyranoside (pNPG), and short-chain fatty acids standards (acetic acid, propionic acid, isobutyric acid, butyric acid, isovaleric acid, valeric acid, and caproic acid) were purchased from Sigma-Aldrich (St.Louis, MO, USA).

### Material preparation and fermentation

Fresh stems of *Dendrobium officinale* (100 g) were rinsed with water and pressed to juice at a 1:4 (w/v) ratio with the water. Then, the sample was sterilized at 121°C for 15 min. After cooling to room temperature, the sample was inoculated with 2% (v/v, 1 × 10^9^ CFU/mL) *Lactobacillus delbrueckii* subspecies *bulgaricus* (GIM 1.155), which was provided by the Guangdong Institute of Microbiology (Guangzhou, Guangdong, China). The sample was placed in a warm air incubator to ferment culture at 37°C for 48 h, and the unfermented sample was used as a control. Lastly, the samples were freeze-dried to obtain crude *Dendrobium officinale* (DO) and fermented *Dendrobium officinale* (FDO) before storing at −20°C until further use.

### *In vitro* gastrointestinal digestion

*In vitro* gastrointestinal digestion was simulated according to the main components of human gastrointestinal digestive juice and digestive environment as described by Xie et al. (23). A human gastrointestinal digestion simulation system is in sequence with healthy gastrointestinal conditions that covered oral, gastric, and small intestinal phases. Firstly, a 4 g sample was mixed with distilled water (45 mL) and simulated saliva fluid stock solution (45 mL, with α-amylase, pH = 6.5) for 2 min under agitation at 37°C. After centrifuging, the supernatant (5 mL) was obtained. After that, the sediment was dispersed with deionized water (40 mL), then simulated gastric fluid (40 mL, with pepsin, pH = 2) was added, and the mixture was stirred for 2 h at 37°C, followed by centrifugation and the supernatant (80 mL) was collected. For the final intestinal phase, the sediment was mixed with distilled water (40 mL) and simulated intestinal fluid stock solution (40 mL, with 800 U/ml pancreatin, and bile salt, pH = 7.0) followed by 2 h incubation at 37°C under constant agitation at 300 rpm. After the digestion was complete, it was centrifuged for 15 min and the supernatant was collected (100 mL). The residues were lyophilized and stored at −80°C prior to the next progress of colon fermentation.

### *In vitro* colonic fermentation

The human fecal fermentation protocols were reviewed and approved by the Academic Committee of Southwest Forestry University (ethical approval number SWFU-2021015). The medium used for fermentation had the following composition (24): 0.005 g FeSO_4_·7H_2_O, 0.08 g CaCl_2_, 0.4 g bile acid, 0.5 g K_2_HPO_4_, 0.69 g MgSO_4_·H_2_O, 0.8 g L-cysteine, 1 g guar gum, 1.5 g NaHCO_3_, 2 g arabinogalactan, 2 g pectin, 3 g casein, 4 g mucoprotein, 4.5 g KCl, 4.5 g NaCl, 4 mL resazurin (0.025%, w/v), and 1 mL Tween 80. The final volume was made up to 1 L with distilled water and then autoclaved for 20 min at 121°C. Fecal samples were obtained from 3 healthy Chinese volunteers (20–25 years old) who had not taken antibiotics at least 6 months before the study and with no history of gastrointestinal disease or surgery. Fresh stool samples (100 g) were obtained from participants at the same time, and then promptly passed into an anaerobic chamber. Immediately, the fecal slurry was prepared by homogenizing fecal samples with 10% (w/v) phosphate-buffered saline (PBS, 0.01 M, pH = 7.2), and was stirred using a sterile glass rod. After filtering the homogenate through two layers of cheesecloth and the filtrate was collected, and stored at −80°C for standby. Eventually, the fecal slurry (50 mL) was mixed in a fermentation medium (50 mL) with the residues (2 g) after gastrointestinal digestion for the experimental groups. The medium with inoculum only was added as the blank group to the plate. Every fermentation step was carried out under anaerobic conditions and continuously stirred at 37°C for 48 h. The reaction was halted by immersing the tubes in an ice bath and collected at 0, 6, 12, 24, and 48 h. The reaction mixtures were then stored at −80°C. The pH of each solution at different time points was measured with a pH meter (Mettler Toledo LE438, China).

### Extraction of phenolic compounds in supernatants

The filtrate (5 mL) of the supernatant collected from each digestion stage was acidified with HCl to pH 1.8–2, and subjected to liquid-liquid extraction with ethyl acetate (3 × 30 mL). The extraction solution was evaporated to dryness at 40°C under reduced pressure. Finally, the volume is made up to 3 mL with ethanol (70%) (23).

### Total phenolic and flavonoid content

The Folin-Ciocalteu method was used to evaluate total phenolic content (TPC) in the different *in vitro* digestion stages (25). Briefly, in a 96-well microplate, the extracts (50 μL) were mixed with 25 μL freshly prepared Folin-Ciocalteu reagent (10%), and 200 μL of sodium carbonate (7.5%, w/v). After 25 min of incubation in the dark, the absorbance was read at 765 nm. Ethanol (70%) was used as a blank sample. All assays were done in triplicate and mean values were averaged. Gallic acid (20–100 μg/mL) was used as the standard curve and results were expressed as milligrams of gallic acid equivalents per gram of dry extract (mg GAE/g DE).

The total flavonoid content (TPC) was estimated using the previously reported method (26). First, 40 μL suitable concentration of sample solution and 15 μL of 5% (w/v) NaNO_2_ were mixed onto a 96-well plate. After 6 min, 20 μL of AlCl_3_ (6%, w/v), 140 μL of NaOH (4%, w/v), and 60 μL of 70% ethanol were added. After incubation for 15 min, the absorbance was determined at 510 nm. Rutin (20–100 μg/mL) was used as standard, and results were expressed as mg of rutin equivalents per g of dry extract (mg RE/g DE).

### The HPLC analysis

The HPLC analysis was performed by an Agilent LC 1260 system (Agilent Technologies, CA, USA) (27). All samples were dissolved in 1,000 μL of methanol and then were filtered by a 0.22-μm membrane. The injection volume was 20 μL. The mobile phase consisted of (A) acetonitrile and (B) water with 0.1% formic acid with the gradient elution programmed as follows: 0 min, 5% A; 5 min, 5% A; 7 min, 10% A; 52 min, 30% A; 65 min, 100% A, following by washing with 100% A for 15 min and re-equilibration of the column with 5% A for 10 min. The chromatogram scan range was set to 200–400 nm. p-Hydroxybenzoic acid, vanillic acid, syringic acid, syringaldehyde, ferulic acid, epigallocatechin gallate, salicylic acid, cinnamic acid, apigenin, and scutellarin were used as standards. The identification was done by comparing the retention time of the corresponding standard at 280 nm, whereas quantification of the compounds was done using five-point calibration curves made by peak area of the standards. The results were expressed as μg per 100 grams of dry extract (μg/100 g DE).

### Antioxidant assay

DPPH radical scavenging activity was assessed as described earlier 22. In total, 100 μL diluted samples solution was mixed with 125 μL of DPPH working solution (0.15 mmol/L) in a 96-well microplate. After 30 min incubation in the dark at room temperature, the absorbance was measured at 517 nm (*A*_*S*_), the 70% (v/v) ethanol solution was used instead of the sample as a negative control (*Ac*), and DPPH% was calculated as the following Equation (1). Trolox (4–20 μg/mL) was used as antioxidant standard curve (y = 3.0857x + 24.15, *R*^2^ = 0.9998) and the radical-scavenging activity of digested and fermented samples was expressed milligram Trolox equivalents per gram of sample dry extract (mg TE/g DE).


(1) DPPH radical scavenging rate (%) = [(Ac − As)/Ac]× 100%

The ABTS radical scavenging activity was measured by a previously described method (28). A 50 μL of the sample with various concentrations was mixed with 200 μL of ABTS·^+^ working solution in a 96-well plate and then the absorbance was measured at 734 nm after 6 min of incubation in the dark at 25°C. ABTS% was calculated using the same equation as Equation (1). The calibration curve was prepared by Trolox (10–60 μg/mL) with the following: y = 1.711x - 2.8472 (*R*^2^ = 0.9907). The final result was expressed as milligrams Trolox equivalent per grams of dry extract (mg TE/g DE).

The antioxidant capacity of samples was also measured by ferric reducing antioxidant power (FRAP) assay (29). A 20 μL of appropriately diluted sample and 300 μL of FRAP fresh solution (10 mmol/L TPTZ, 20 mM FeCl_2_, and 300 mM acetate buffer were prepared in the ratio of 1:1:10) were mixed in a 96 well plate. The absorbance was read at 593 nm using a microplate reader after incubation for 10 min at 37°C. FRAP values were calculated using a standard curve (y = 0.0084x - 0.0343, *R*^2^ = 0.991) of FeSO_4_ (4–20 μg/mL), which were expressed in terms of milligram of FeSO_4_ per gram of dry extract (mg FeSO_4_/g DE).

### α-Amylase inhibitory activity assay

The α-amylase inhibitory activity was determined by the dinitrosalicylic acid (DNS) method (30). First, a 20 μL properly diluted sample solution and 20 μL of α-amylase (0.1 U/mL, pH 6.9) were added into a 96-well plate and then incubated at 37°C for 10 min. Subsequently, 40 μL soluble starch (0.25%, w/v) was added and the reaction mixture was incubated at 37°C for 5 min, followed by the immediately adding of 20 μL 3,5-dinitrosalicylic acid (DNS) color reagent to stop the reaction. Finally, the micro-well plates were placed in a boiling water bath for 10 min. After cooling at room temperature, 100 μL distilled water was added, and the absorbance was measured at 540 nm (*As*). The groups without the α-amylase (*Ac*) and without the samples (*Ab*) were used as a negative control and blank control, respectively. The α-amylase inhibitory activity was calculated according to Equation (2). Acarbose (20–200 μg/mL) was used served as the standard inhibitor to plot curve (*y* = 0.003x + 0.2013, *R*^2^ = 0.9944), and results were expressed as milligram acarbose per grams of dry extract (mg acarbose/g DE).


(2) α-Amylase inhibitory (%) = [(As − Ac)/Ab]×100

Where, *As* were the absorbance value of the reaction system with samples, *Ab* and *Ac* were the absorbance of blank and control, respectively.

### α-Glucosidase inhibitory activity assay

The α-glucosidase inhibitory activity was measured based on the slightly modified method (31). 50 μL of suitably diluted sample and 50 μL of α-glucosidase solution (1 U/mL, pH = 6.9) were incubated on 96-well plates at 25°C for 5 min. After that, 50 μL of 5 mM p-nitrophenyl-α-d-glucopyranoside (p-NPG) solution was mixed to incubate at 25°C for 25 min. Finally, 100 μL of 0.2 M Na_2_CO_3_ solution was added to stop the enzymatic reaction, and the absorbance at 405 nm was measured (*As*). Seventy percent ethanol was used as the blank (*Ab*) and a group without the α-glucosidase as the negative control (*Ac*). The inhibitor drug acarbose (20–100 μg/mL) was used as reference standard (y = 0.0043x + 0.0845, *R*^2^ = 0.9935), and the inhibition ability was expressed as milligram acarbose per grams of dry extract (mg acarbose/g DE). The inhibition rate was calculated using Equation (2).

### SCFA analysis

Gas chromatography-mass spectrometry (GC-MS) analysis of SCFAs composition and production in the fecal samples was performed as previously described (32). Briefly, 2 mL of sample was thawed at room temperature, followed by addition of 50 μL of phosphoric acid (w/v%, 5%), a 100 μL of isocaproic acid solution (125 μg/mL) and 400 μL diethyl ether were then added to homogenate on ice for 1 min. Finally, the supernatants were collected following centrifugation at 12,000 rpm for 15 min at 4°C for further GC-MS analysis. MS condition: an electron bombardment ion (EI) source (70 eV), and the data collection method was selective ion monitoring (SIM) mode. Helium was the carrier gas with a flow rate of 1 mL/min. All injections were 1 μL with a split injection ratio of 10:1. The capillary GC-MS column is HP-Innowax (30 m × 0.25 mm × 0.5 μm, Agilent Technology, USA). The ion source temperature was set at 200°C, and the injection port and transfer line temperature were kept at 250°C. The GC temperature program: start with an initial temperature of 90°C, after which the column was increased to 120°C at a rate of 10°C/min, followed by 150°C at a rate of 5°C/min, and finally 10 min at 250°C (maintained for 2 min).

### 16S rRNA gene sequencing analysis

Total genomic DNA from samples was extracted using the previously method (33). DNA concentration and purity were detected with 1 ng/μL sterile water. PCR amplification was performed at the variable region (V3-V4) of the 16S rRNA genes using Barcarole specific primers and High-Fidelity DNA Polymerase. The forward primer was 341F (CCTAYGGGRBGCASCAG), and the reverse primer was 806R (GGACTACNNGGGTATCTAAT0). The PCR products were mixed with an equal volume of 1 × loading buffer (containing SYB green) and were examined by 2% agarose gel electrophoresis for detection. Then, the mixture of PCR products was purified by using AxyPrepDNA Gel Recovery Kit (Axygen, China). Referring to the preliminary quantitative results of electrophoresis, the PCR products were quantified with a QuantiFluor™-ST blue fluorescence quantitative system (Promega, United States). The library quality and average fragment sizes were assessed on the Agilent Bioanalyzer 2100 system and Qubit@ 2.0 Fluorometer (Thermo Scientific). Subsequently, the libraries were sequenced with Illumina Miseq/HiSeq2500 and 250/300 bp paired-end reads were generated.

Paired-end reads were assigned to each sample based on their unique barcode, which was used for bioinformatic processing. Sequences with ≥ 97% similarity were assigned to the same Operational Taxonomic Units (OTUs), and the richness and species of Alpha diversity were assessed. Linear discriminant analysis effect size (LEfSe) was conducted for quantitatively analyzing biomarkers in different groups, and KEGG pathway enrichment analysis was performed.

### Statistical analyzes

Each experiment was performed at least three times, and all numerical data are expressed as the mean ± standard deviation. Statistical analyses were performed using Origin 2018 software, and significant differences among the groups were analyzed using SPSS Version 22.0. Correlations were determined by the Pearson correlation coefficient. Differences in operational taxonomic units (OTUs) were evaluated using the raw data reads. LEfSe was performed using the LEfSe tool (https://www.omicstudio.cn/tool/60). The least significant difference (LSD) test was adopted to analyze the significant differences among multiple groups. The KEGG analysis was performed by KEGG Mapper (http://www.genome.jp/kegg/mapper.html).

## Results

### Total phenolic and flavonoid contents analysis

The changes in the TPC and TFC of DO and FDO during the simulation of gastrointestinal digestion and colonic fermentation are shown in Table 1. For DO, the undigested control sample contained approximately 2.16 ± 0.26 mg GAE/g DE of TPC, and no significant change was found in the oral phase (2.64 ± 0.11 mg GAE/g DE), while there were substantial increases in the gastric and intestine phases with values of 3.74 ± 0.19 and 5.74 ± 1.06 mg GAE/g DE (*p* < 0.05), respectively. The release of TPC in FDO showed a similar trend for DO. The intestine phase was the highest (6.96 ± 0.99 mg GAE/g DE), which was 2.48-fold of the undigested phase (2.80 ± 0.14 mg GAE/g DE), followed by the gastric phase (4.40 ± 0.29 mg GAE/g DE), and the oral phase (3.01 ± 0.24 mg GAE/g DE). After fermentation in the colonic for 24 h, both for DO and FDO, the released amount of TPC increased slightly, and declined thereafter, but not significant (*p* > 0.05).

**Table 1:** TPC and TFC values of DO and FDO *in vitro* gastrointestinal digestion and colonic fermentation.

			***In vitro*** **digestion**			**Colonic fermentation**			
		**Undigested**	**Oral**	**Gastric**	**Intestine**	**6 h**	**12 h**	**24 h**	**48 h**
TPC	DO	2.16 ± 0.26^c^	2.64 ± 0.11^c^	3.74 ± 0.19^b^	5.74 ± 1.06^a^	2.25 ± 0.12^c^	2.29 ± 0.10^c^	2.81 ± 0.04^c^	2.73 ± 0.05^c^
	FDO	2.80 ± 0.14^c^	3.01 ± 0.24^c^	4.40 ± 0.29^b^	6.96 ± 0.99^a*^	2.78 ± 0.04^c^	2.80 ± 0.10^c^	2.96 ± 0.17^c^	2.84 ± 0.12^c^
TFC	DO	2.14 ± 0.34^b^	2.51 ± 0.62^a, b^	3.39 ± 0.62^a^	3.48 ± 0.85^a^	2.16 ± 0.40^b^	2.01 ± 0.28^b^	2.45 ± 0.39^ab^	1.70 ± 0.46^b^
	FDO	6.84 ± 0.34^c*^	8.56 ± 0.58^b*^	10.61 ± 0.35^a*^	10.70 ± 1.31^a*^	2.64 ± 0.13^d^	2.65 ± 0.48^d^	2.80 ± 0.26^d^	2.50 ± 0.37^d^

TPC, total phenolic content (mg GAE/g DE); TFC, total flavonoid content (mg RE/g DE); “DO” and “FDO” indicated the *Dendrobium officinale* and fermented *Dendrobium officinale*, respectively. ^a−*d*^Mean values in the same row indicated the significantly different (*p* < 0.05), * Mean values indicated the significantly different in the same indicator at the same time point between DO and FDO (*p* < 0.05).

Similar to TPC, the gastrointestinal phase also led to a substantial increase in TFC of the DO and FDO. Of which, the TFC of the undigested DO was found to be 2.14 ± 0.34 mg RE/g DE, and there was no significant change after oral digestion (2.51 ± 0.62 RE/g DE). While in gastric and intestine, their TFC were 3.39 ± 0.62 and 3.48 ± 0.85 RE/g DE, which was a statistically significant increase of 58 and 62%, respectively, in comparison with the undigested control (*p* < 0.05). During the colonic fermentation stage, the TFC also first increased and then decreased, and it was highest after colonic fermentation for 24 h (2.45 ± 0.39 RE/g DE). When compared to DO, FDO demonstrated the surprising TFC values: 6.84 ± 0.34 mg RE/g DE for the undigested sample, 8.56 ± 0.58 mg RE/g DE for the oral phase, 10.61 ± 0.35 mg RE/g DE for the gastric phase, and 10.70 ± 1.31 mg RE/g DE for the intestine phase. The TFC values of FDO were even 3.2 times higher than that of DO during the whole digestion. A similar trend was observed for the TFC in colonic fermentation with that of DO, and the highest value was found after colonic fermentation for 24 h (2.80 ± 0.26 RE/g DE).

Overall, during digestion simulation, there was a progressive increase in the TPC and TFC of both DO and FDO, which means that the digestion could promote the release of phenols and flavonoids. Bound phenolic compounds were conjugated with macromolecules such as proteins, carbohydrates, and pectin through covalent bonds and non-covalent bonds before being released from the food matrix by various digestive enzymes and acid-base environments in intestine time, and the remaining phenolics will then directly move to the colon (34). The TPC and TFC of DO and FDO reached the highest peak at 24 h colonic fermentation, it was probably demonstrated that a large number of polyphenols bound with DO or FDO were subjected to colonic fermentation, then slowly and continuously released under the influence of gut microbe (24). The colon fermentation decreases after 24 h, which may be because some of the bound polyphenols could be degraded and transformed as the activity of microbe present in the human gut (35). On the other hand, the FDO almost had higher TPC and TFC values than DO nearly for each phase (*p* < 0.05), which indicated fermentation could also significantly increase the release of the phenols and flavonoids in DO, and, subsequently, promotes the bioaccessibility of PPs. The reason might be that probiotic fermentation could consume the macromolecules bound with phenolics or flavonoid, which led to the release of phenolics and flavonoid (36).

### HPLC analyzes

**Figure 1:**
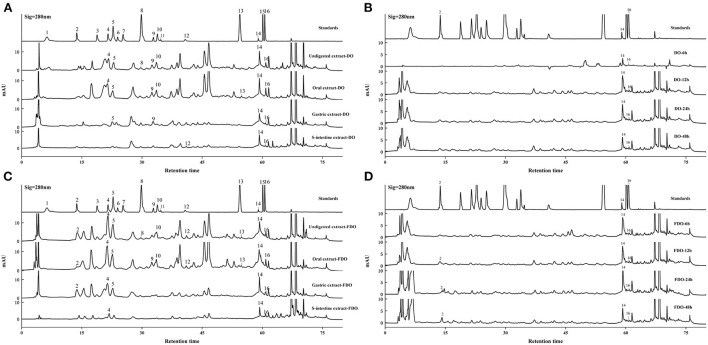
HPLC-DAD chromatogram of DO **(A,B)** and FDO **(C,D)** *in vitro* gastrointestinal digestion and colonic fermentation. mAU: milli-arbitrary units. Detection at 280 nm: (1) Gallic acid, (2) P-hydroxybenzoic acid, (3) Protocatechuic acid, (4) Vanillic acid, (5) Syringic acid, (6) Epicatechin, (7) Dihydromyricetin, (8) Syringaldehyde, (9) Ferulic acid, (10) Epigallocatechin gallate, (11) Ellagic acid, (12) Salicylic acid, (13) Cinnamic acid, (14) Apigenin, (15) Hesperetin, (16) Scutellarin.

The contents of phenolic compounds of DO and FDO before and after *in vitro* digestion and colonic fermentation are tabulated in Table 2. According to the previous studies, 16 standards were selected (37–39). These standards and compounds were identified and quantified using HPLC-DAD by comparing the retention times and peak area with their authentic standards. The chromatograms of the samples are illustrated in Figure 1. A total of six phenolic acids and four flavonoids were identified in the DO and FDO during *in vitro* digestion. To begin with, we have detected seven phenolics including vanillic acid, syringic acid, syringaldehyde, ferulic acid, epigallocatechin gallate, apigenin, and scutellarin in the undigested DO. In the undigested phase, the contents of phenolic compounds of FDO were generally higher than that of DO except for ferulic acid and epigallocatechin gallate, while three new phenolic acids were emitted in FDO: p-hydroxybenzoic acid, salicylic acid, and cinnamic acid. For the oral phase, the content of PPs were enhanced significantly compared to that of the undigested one, the released salicylic acid (11.21 ± 3.90 mg/100 g DE) and apigenin (11.22 ± 3.91 mg/100 g DE) from FDO were about 3.4 and 1.8 times higher than the undigested FDO, respectively.

**Table 2:** The content of individual phenolics from DO and FDO released *in vitro* gastrointestinal digestion.

**Compounds**	**Classification**	**Undigested**		***In vitro*** **digestion**					
**(mg/100g DE)**		**DO**	**FDO**	**DO-oral**	**FDO-oral**	**DO-gastric**	**FDO-gastric**	**DO-intestine**	**FDO-intestine**
P-hydroxybenzoic acid	Phenolic acids	ND	1.07 ± 0.05^c^	ND	1.88 ± 0.08^a^	ND	1.34 ± 0.32^b^	ND	ND
Vanillic acid	Phenolic acids	2.60 ± 0.09^e^	3.19 ± 0.03^d^	2.65 ± 0.07^e^	3.37 ± 0.11^c^	ND	5.25 ± 0.06^a^	ND	4.88 ± 0.07^b^
Syringic acid	Phenolic acids	0.65 ± 0.06^c^	1.14 ± 0.06^b^	0.72 ± 0.02^c^	1.18 ± 0.12^b^	0.17 ± 0.01^d^	1.55 ± 0.04^a^	ND	ND
Syringaldehyde	Flavonoids	0.10 ± 0.03^b^	0.34 ± 0.02^a^	ND	ND	ND	ND	ND	ND
Ferulic acid	Phenolic acids	0.42 ± 0.06^c^	ND	0.79 ± 0.04^b^	0.95 ± 0.20^b^	1.33 ± 0.09^a^	ND	ND	ND
Epigallocatechin gallate	Flavonoids	2.92 ± 0.25^a^	0.47 ± 0.02^b^	0.46 ± 0.04^b^	0.59± 0.09^b^	ND	ND	ND	ND
Salicylic acid	Phenolic acids	ND	3.29 ± 0.20^b^	ND	11.21 ± 3.90^a^	ND	ND	3.70 ± 0.03^b^	ND
Cinnamic acid	Phenolic acids	ND	0.23 ± 0.00^b^	0.28 ± 0.01^b^	0.40 ± 0.00^a^	ND	ND	ND	ND
Apigenin	Flavonoids	5.73 ± 1.24^c^	6.17 ± 1.94^c^	6.10 ± 0.44^c^	11.22 ± 3.91^ab^	6.61 ± 0.77^c^	13.27 ± 1.28^a^	9.15 ± 1.41^bc^	7.53 ± 0.41^c^
Scutellarin	Flavonoids	0.37 ± 0.01^d^	0.38 ± 0.01^d^	0.43 ± 0.02^c^	0.41 ± 0.00^c^	0.74 ± 0.01^d^	0.71 ± 0.01^b^	0.71 ± 0.01^b^	0.70 ± 0.02^b^

“DO” and “FDO” indicated the *Dendrobium officinale* and fermented *Dendrobium officinale*, respectively. ^a−*e*^Letters in row indicated the significantly different (*p* < 0.05), ND, not detected.

Moreover, a new phenolic compound of cinnamic acid (0.28 ± 0.01 mg/100 g DE) was released in the DO during the oral phase, perhaps because amylase could enhance the release of phenolics from the food matrix. Interestingly, the majority of PPs were decreased stepwise from oral to gastric digestion, and even less in the intestinal phase, but some PPs were released after gastrointestinal digestion. In gastric digestion compared with oral digestion, the released ferulic acid, apigenin, and scutellarin in DO were increased by 68.3, 8.3, and 72%, respectively, while the released syringic acid decreased. However, ferulic acid, epigallocatechin gallate, salicylic acid, and cinnamic acid in FDO of the gastric phase were not detected, whereas the concentrations of vanillic acid, apigenin, and scutellarin in the gastric phase were slightly increased, respectively, 1.31, 1.18, and 1.73 folds of that in oral phase. For the DO sample, only apigenin and scutellarin were released at the end of the intestinal phase, and the intestinal phase was the only process seen to promote the release of salicylic acid. While for the FDO sample, the contents of apigenin and scutellarin were lower than that of DO in the intestinal, and they were present throughout the *in vitro* gastrointestinal digestion. Surprisingly, vanillic acid was detected only in FDO at the intestinal phase (4.88 ± 0.07 mg/100 g DE). It presumably because of the release degree of PPs during digestion was greatly affected by alkaline and acidic conditions, flavonoids have been reported to be more stable under acidic conditions, however, in neutral or alkaline environments, such as in the intestine, they degrade which produces simpler phenolic acids (40).

**Figure 2:**
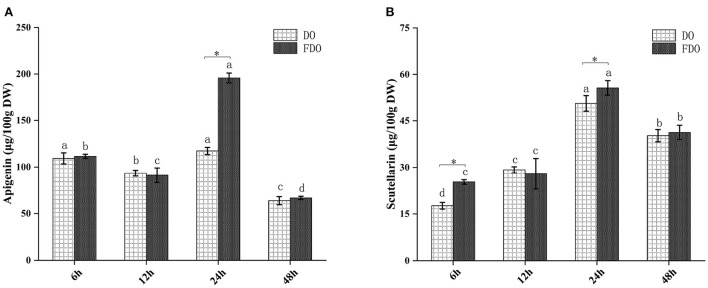
The content of apigenin **(A)** and scutellarin **(B)** from DO and FDO at different time point of colonic fermentation. DO, *Dendrobium officinale*; FDO, fermented *Dendrobium officinale*. Means with different letters are significantly different (*p* < 0.05), * Mean values indicated the significantly different in the same indicator at the same time point between DO and FDO (*p* < 0.05).

The release of these active compounds during colon fermentation was shown in Figure 2. Both for DO and FDO, the contents of apigenin and scutellarin were all the highest at 24 h of colonic fermentation, where FDO were respectively 1.36 and 1.2 times greater than DO. Moreover, compared with DO, p-hydroxybenzoic acid was only released in FDO during colon fermentation, which might be due to the action of *Lactobacillus* fermentation, causing the release or production of new compounds.

### Antioxidant activity

**Figure 3:**
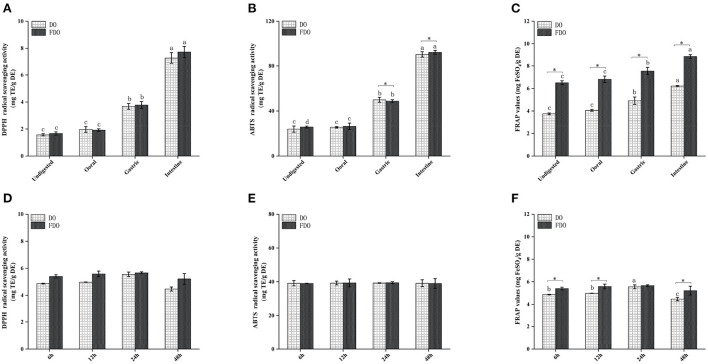
Antioxidant activity of DO and FDO during *in vitro* digestion and colonic fermentation processing. **(A,D)** DPPH radical scavenging activity, **(B,E)** ABTS radical scavenging ability, **(C,F)** Ferric reducing antioxidant power. DO, *Dendrobium officinale*; FDO, fermented *Dendrobium officinale*. Means with different letters are significantly different (*p* < 0.05), * Mean values indicated the significantly different in the same indicator at the same time point between DO and FDO (*p* < 0.05), while no letters mean no significant difference (*p* > 0.05).

Several beneficial effects of PPs have been attributed to their antioxidant capacity as well as their stability and availability during the digestion process. Thus, we further investigated the effect of digestion on the antioxidant capacities of DO and FDO PPs along the *in vitro* simulated gastrointestinal digestion. Antioxidant capacities were measured by the DPPH and ABTS radical scavenging activity, and ferric reducing antioxidant power (Figure 3). The DPPH values during the intestinal digestion phase in the FDO were up to 4.67, 4.40, and 2.04 times higher than those measured for the undigested, oral, and gastric digestion phase, respectively (Figure 3A). From Figure 3D, the FDO after 6 h to 24 h of colonic fermentation showed increased DPPH radical scavenging capacity of 3.10 ± 0.42 to 3.21 ± 0.46 mg TE/g DE, and further decreased to 3.06 ± 0.20 mg TE/g DE after 48 h. In addition, the DPPH radical scavenging activity of FDO overall was also stronger than that of the DO in digestion and colonic fermentation.

Similar behavior was observed when ABTS was evaluated (Figure 3B). The intestinal digestion product of the FDO processed by fermentation displayed the highest ABTS scavenger radical capacity (92.37 ± 1.76 mg TE/g DE) followed by gastric digestion (48.71 ± 0.25 mg TE/g DE) and oral digestion (26.36 ± 3.86 mg TE/g DE), which was higher than that of undigested fractions with 25.74 ± 0.83 mg TE/g DE. In terms of ABTS scavenging activity after colonic fermentation, DO and FDO samples showed a steady trend with no significant difference (Figure 3E, *p* > 0.05). This is understandable because the number of PPs was not only lesser in the colonic fermentation stage but also reduced in the content.

After undergoing the oral phase compared to those undigested, a significantly larger FARP value was observed in the DO and FDO samples (Figure 3C), with values of 4.06 ± 0.08 and 6.81 ± 0.28, respectively. The gastric phase was more striking, with an increase in FRAP of DO and FDO of 16 and 10%, respectively. In the intestinal phase, the FRAP values of DO and FDO maintained the increasing trend and reached a maximum of 6.21 ± 0.04 mg FeSO_4_/g DE and 8.86 ± 0.15 mg FeSO_4_/g DE, respectively. Additionally, for each DO and FDO sample, there were significant differences in the FRAP colonic fermentation phase (Figure 3F, *p* < 0.05), and the values of FRAP were 4.81–5.59 and 5.3–5.64 mg FeSO_4_/g DE, respectively. Interestingly, the FRAP values of FDO are overall significantly stronger than DO (*p* < 0.05). In summary, a comparison of the DO and FDO groups revealed that the intestinal phase had elevated significant antioxidant activity when compared with the non-digested ones (*p* < 0.05) during the entire digestion. It might be relevant to the release content, biological activity, stability, and bioavailability of PPs after digestion. The same finding occurred in the gastric and intestinal digestion process of apple and mango fruit, which showed higher polyphenol content and antioxidant activities after the *in vitro* digestion (41, 42).

**Figure 4:**
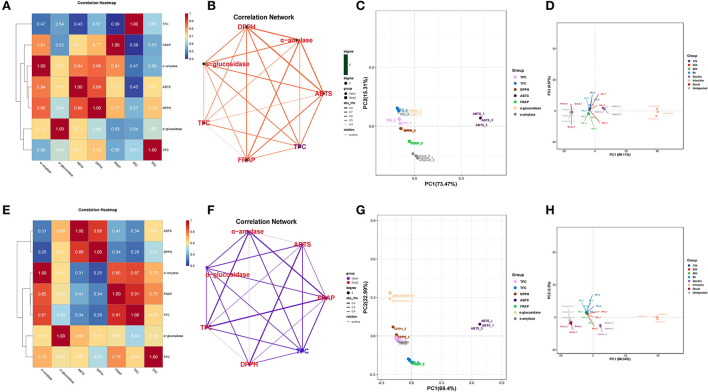
Heatmap graph of correlation analysis and principal component analysis (PCA) of different samples and indicators. **(A,E)** Correlation heatmap of DO and FDO. **(B,F)** Correlation network of DO and FDO. **(C,G)** Principal coordinates analysis (PCoA) of 7 functional activity indicators in DO and FDO; **(D,H)** Principal component analysis of different digestion stages in DO and FDO. *P*-value (ranging from 0 to 1) and corresponding color (red to blue) represented the magnitude of the Pearson correlation. TFC, total flavonoid content; DPPH, DPPH· scavenging activity; ABTS, ABTS·+ scavenging ability; FRAP, ferric reducing antioxidant power; α-amylase, α-amylase inhibitory; α-glucosidase, α-glucosidase inhibitory.

The correlation analysis showed good correlations between the DPPH and ABTS with TPC in DO (Figure 4A), and the correlation coefficients were 0.71 and 0.74, respectively. Significant positive correlations were also found FRAP with TFC (*r* = 0.87, *p* < 0.01) in FDO (Figure 4E), which were even higher than their correlations with TPC. In all, a linear relationship was found between the TPC/TFC and functional activity indicators that were positively correlated to different degrees in the digestion, which suggested that the content of released PPs contributed significantly to the antioxidant capacity of the DO and FDO. To exclude the effects of other factors on the results, we further plotted the principal coordinates analysis (PCoA) about seven functional activity indicators (Figures 4C,E) and principal component analysis (PCA) about different digestion stages in DO and FDO (Figures 4D,H), the results exhibited that each group can be well-differentiated.

### α-Amylase and α-Glucosidase inhibitory ability

**Figure 5:**
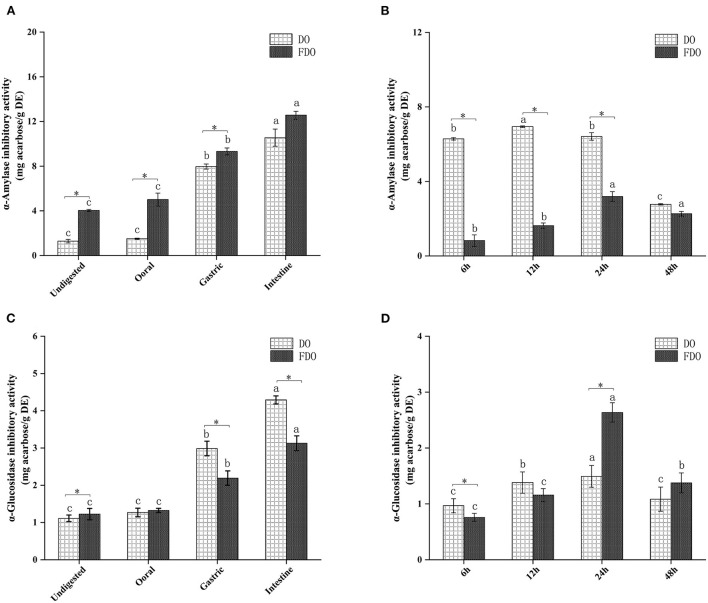
The α-amylase **(A,B)** and α-glucosidase **(C,D)** inhibitory activities of released from DO and FDO after *in vitro* gastrointestinal digestion and colonic fermentation. DO, *Dendrobium officinale*; FDO, fermented *Dendrobium oficinale*. Means with different letters are significantly different (*p* < 0.05), * Mean values indicated the significantly different in the same indicator at the same time point between DO and FDO (*p* < 0.05).

Inhibition of α-amylase and α-glucosidase enzymes delays the digestion of carbohydrates and glucose absorption in the small intestine, resulting in reduced postprandial plasma glucose levels, thereby delaying postprandial hyperglycemia (43). In the last few years, various α-glucosidase inhibitors from natural products have been widely used as they are safe and have minimal side effects (44). Acarbose is the drug that is most commonly and extensively used as α-amylase and α-glucosidase inhibitors (45), hence, the enzyme inhibition potency in this study was indicated by mg acarbose/g DE. The α-amylase inhibitory activity of DO and FDO at different phases of *in vitro* gastrointestinal digestion (Figures 5A,C) and colonic fermentation (Figures 5B,D) were measured. The α-amylase inhibitory activity of FDO was higher than that in DO during the whole digestion process, and the inhibitory activity of FDO was primarily shown in the small intestine, which reached the highest (15.43 ± 0.27 mg acarbose/g DE), but there was no significant difference between DO and FDO at this phase (*p* > 0.05). Nevertheless, Figure 5B shows that the α-amylase inhibitory activity of DO was significantly higher than the FDO with colonic fermentation at 6 h to 48 h, indicating some bioactive substances in DO at this stage were involved in controlling α-amylase activity.

The inhibitory activities of α-glucosidase showed no significant difference among the DO and FDO (*p* > 0.05) at the oral digestion phase, indeed the DO in most stages of gastrointestinal digestion has significantly higher inhibitory effects against α-glucosidase than FDO (*p* < 0.05), and illustrated the strongest α-glucosidase inhibitory activity in the intestinal phase, with the value of 4.29 ± 0.3 mg acarbose/g DE. However, all samples showed a weaker α-glucosidase inhibitory activity after colonic fermentation when compared with those of intestinal digestion. It was interesting to note that DO has shown a higher inhibition of α-glucosidase (*p* < 0.05) than FDO at 6 h of colonic fermentation. Surprisingly, the α-glucosidase inhibitory activity of FDO drastically increased and even reached the highest at 24 h (2.63 ± 0.17 mg acarbose/g DE) during the progress of colonic fermentation, and relatively declined at 48 h (1.37 ± 0.17 mg acarbose/g DE).

**Figure 6:**
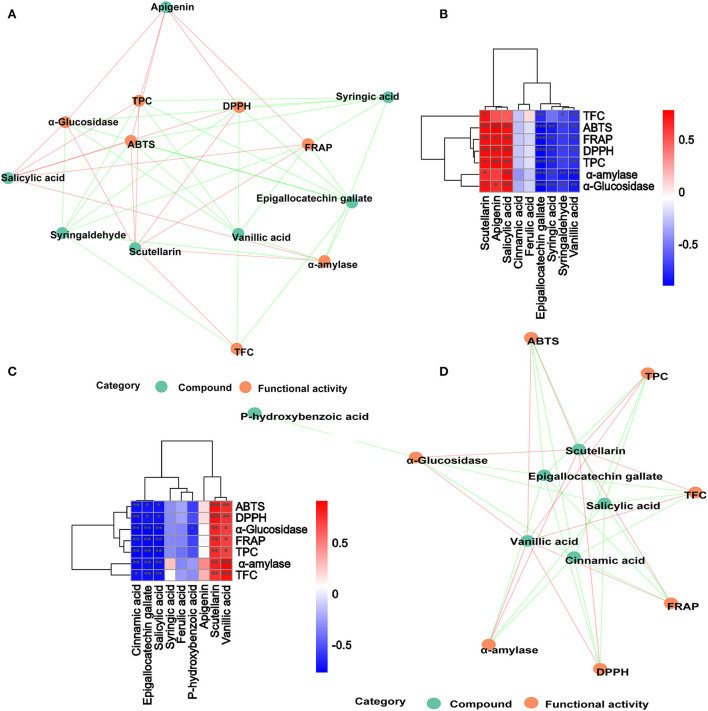
Associated network diagram and correlation heat map of spearman's analysis shows the correlation between metabolites and functional activities. **(A,C)** Associated network of DO and FDO. **(B,D)** Correlation heat map of spearman's analysis of DO and FDO. Red lines represent positive correlations while green lines represent negative correlations between compounds (green circle) and functional activities (red circle). *p < 0.05, **p ≤ 0.01, and ***p < 0.001, respectively.

In order to further analyze the correlations among 10 metabolites and seven functional activities in DO and FDO, the correlations were evaluated by Spearman's method and displayed as network diagrams and heat maps (Figure 6). For DO, three PPs of scutellarin, salicylic acid and apigenin were significantly positively correlated with TFC, antioxidant activity and α-glucosidase inhibitory ability (*p* < 0.05), while four metabolites (syringic acid, syringaldehyde, epigallocatechin gallate, and vanillic acid) showed significantly negatively correlated with all activity indicators. An unexpected finding of this study was that compared with DO, only vanillic acid and scutellarin in FDO were significantly positively correlated with all functional activity indicators (*p* < 0.05). Apigenin in FDO was not significantly correlated with α-glucosidase inhibitory ability, but there was a significant positive correlation in DO, which explained the stronger inhibition of glucosidase by DO than FDO during the intestinal phase. The network diagrams demonstrated that the complex relationships and interaction between the abundant metabolites and functional activities indicators for DO (Figure 6A) and FDO (Figure 6D), a functional index may be regulated by a variety of active substances.

### Effects of DO and FDO on pH values in colonic fermentation

**Figure 7:**
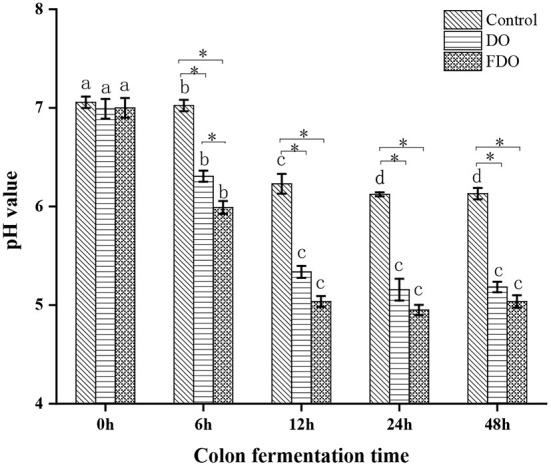
The changes in pH values of fermented cultures added with DO or FDO at different time point of 0, 6, 12, 24, and 48 h. DO, *Dendrobium officinale*; FDO, fermented *Dendrobium officinale*. Means with different letters are significantly different (*p* < 0.05), *Mean values indicated significantly different (*p* < 0.05).

A human fecal inoculum simulated fermentation model was used to assess the performance of DO and FDO. During the colonic fermentation, the pH values are an important factor, which can reflect the fermentation process and degree. From Figure 7, the initial pH values for all groups were approximately 6.99 to 7.05, then reached 6.30 for DO and 5.99 for FDO after fermentation for 6 h, while the control group remained 7.02. When the fermentation time was 12 h, these pH values significantly decreased to 5.33, 5.03, and 6.23, respectively (*p* < 0.05). Then, that continuously declines until it stabilizes at fermentation for 24 h. Altogether, the pH value of the colonic culture significantly changed over time, of which the FDO group was lower during the entire fermentation, and demonstrated the highest decrement (from 7.0 to 5.0) when compared to DO and control groups (*p* < 0.05). The results indicated that DO and FDO could decrease the pH of the to the contents of colonic fermentation, with FDO being more potent. The decrease in colonic pH might be related to the consumption of microbial carbon sources during fermentation and the production of organic acids (46).

### Effects of DO and FDO on SCFAs in colonic fermentation

**Figure 8:**
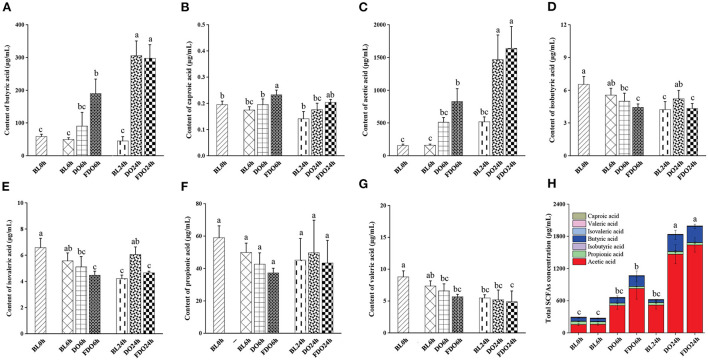
Concentrations of butyric acid **(A)**, caproic acid **(B)**, acetic acid **(C)**, isobutyric acid **(D)**, isovaleric acid **(E)**, propionic acid **(F)**, valeric acid **(G)**, and total SCFAs **(H)** in fermentation products of DO and FDO compared to blank group. Different letters are used to denote mean values that are significantly different between treatments (*p* < 0.05).

SCFAs, as a major class of key microbial metabolic products in the large intestine and colon, is an important indicator of intestinal homeostasis and have a significant relation to human health. In addition, bacterial fermentation dietary fibers may lower the pH of the colon through the production of SCFAs, which influence the fermentation environment of the colon. According to Section 4.5, the pH value maintained a decreased trend after colon fermentation for 6 h and then remained stable at 24 h, therefore we chose the colon fermentation 6 h and 24 h for SCFAs analysis. A total of 7 samples were divided into fermentation 0 h (BL0h), fermentation 6 h (BL6h, DO6h, and FDO6h), and fermentation 24 h (BL24h, DO24h, and FDO24h). Based on this, seven SCFAs and total SCFAs in these samples were subsequently investigated, including butyric, caproic, acetic, isobutyric, isovaleric, propionic, and valeric acids. From Figure 8, the amounts of SCFAs produced *in vitro* fecal fermentation with DO and FDO are generally higher than those of the control without them. The fermentation of 24 h in group FDO produced significantly higher concentrations of acetic acid (517.94 ± 74.33 μg/mL) and butyric acid (305.61 ± 6.53 μg/mL) compared with the blank (*p* < 0.05). At the same time, the fermentation of 6 h and 24 h in DO groups generated more acetic acid, propionic acid, and total SCFAs than blank, but remained substantially below FDO groups. Furthermore, the concentrations of caproic, isobutyric, isovaleric, propionic, and valeric acid were not notably increased in the fermentation of all groups (*p* > 0.05).

Among these seven SCFAs, the total content of acetic acid and butyric acid represent 90–95% of the total SCFAs present in the colon, another five SCFAs comprise 5% of the total. Compared with the other six acids, higher amounts of acetic acids were produced in fecal fermentation with DO and FDO, suggesting the change in the pH is mainly affected by acetic acid. Previous studies have suggested that acetic acid showed bactericidal activity, which inhibited the number of harmful bacteria, thereby maintaining the balance of gut flora (47). This finding suggested that DO and FDO, especially FDO, might have beneficial effects on human gut health through promoting acetic acid production.

### Effect of DO and FDO on fecal microbial

**Figure 9:**
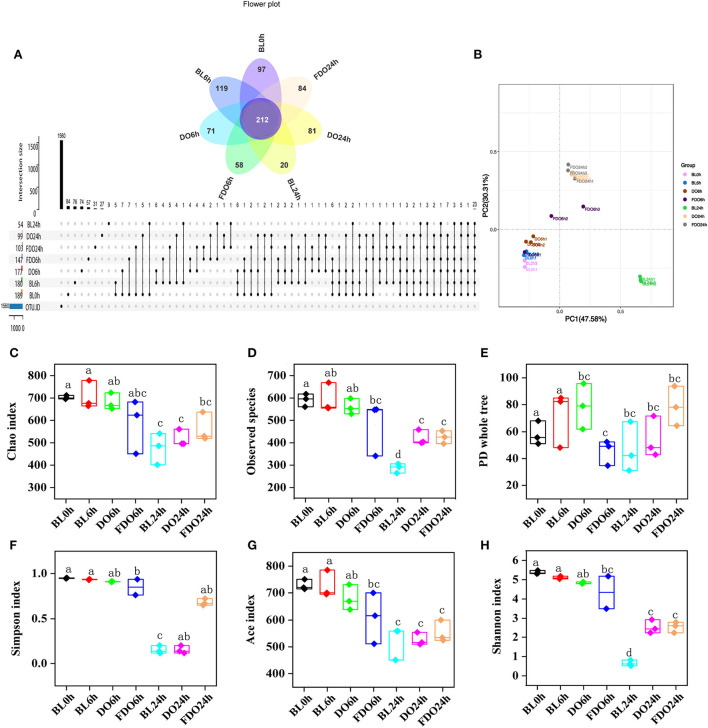
Alpha diversity indices boxplot among groups. **(A)** Upset plot and petal diagram; **(B)** PCoA analysis; **(C)** Chao index; **(D)** Observed species; **(E)** PD whole tree; **(F)** Simpson index; **(G)** ACE index; **(H)** Shannon index; BL, group of fermentation *in vitro* without DO and FDO; DO, *Dendrobium officinale*; FDO, fermented *Dendrobium officinale*. Boxes with different letters are used to denote mean values that are significantly different (*p* < 0.05).

The species and number of the gut microbiota were reported to correlate with some of the diseases in human health (48). To assess the effect of DO and FDO on fecal bacterial microbiota, 16S rRNA high throughput sequencing was further used to analyze the diversity and change in the composition of human fecal microbial community during colon fermentation. In general, sequences with at least 97% similarity were clustered into OTUs, which can represent the community richness. The same 7 samples as in Section 4.6 were carried out for OTUs analysis, and there were 189, 180, 177, 147, 54, 99, and 103 OTUs for the BL0h, BL6h, DO6h, FDO6h, BL24h, DO24h, and FDO24h, respectively (Figure 9A). There was a steady decrease in the OTU richness of the entire community as the fermentation time increased, of which the number of OTUs for the FDO24h was more than BL24h. The principal coordinate analysis (PCoA) plots using weighted distance were performed and a clear separation between each experimental group was observed (Figure 9B). According to the alpha diversity analyses (Figures 9C,D), colon fermentation could significantly alter the microbiota profile of the fecal microbial community, and decrease its diversity and richness. Interestingly, both DO and FDO could improve the diversity and richness of fecal microbial community, and the effect of FDO at colon fermentation 24 h was more obvious. In short, ingestion of FDO that more effectively improved gut flora diversity.

**Figure 10:**
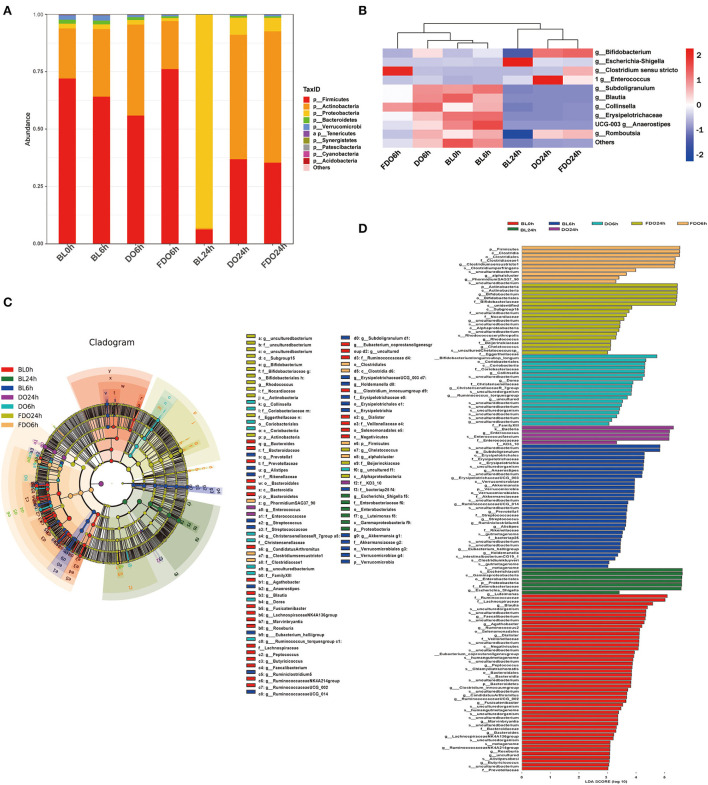
Effects of DO and FDO on the structure of the gut microbiota composition *in vitro* fecal fermented at different times. **(A)** Microbial composition at phylum level during *in vitro* fecal fermentation; **(B)** The heatmap of the gut microbiota in all groups at the genus level; Histogram **(C)** and cladogram **(D)** from LEfSe analysis. BL, group of fermentation *in vitro* without DO and FDO; DO, *Dendrobium officinale*; FDO, fermented *Dendrobium officinale*.

At the phylum level, Firmicutes, Proteobacteria, Actinobacteria, and Bacteroidetes were the main phyla for each group (Figure 10A). Of which, the BL0h group was dominated by Firmicutes and Actinobacteria. After fermentation for 24 h, the concentrations of Proteobacteria in all groups were increased. In the BL24h group, Proteobacteria became the dominant flora with a proportion of 92.89%. Compared with BL0h, a relative increase in abundance of Firmicutes in the DO24h (by 30.58%) and in the FDO24h (by 29.12%). Similarly, there were exhibited a large increase relative abundance of Actinobacteria in the DO24h and FDO24h (by 53.54 and 56.49%, respectively). In contrast, fermentation in colon for 24 h, a significant reduction was observed for Proteobacteria with a decrease rate of 85.44% in DO and 87% in FDO (*p* < 0.05). This was consistent with the results reported previously (49).

The overall microbiota structure for each group at the genus level is shown in Figure 10B as a heat map. For the blank sample, *Escherichia-Shigella*, one of the most common pathogenic bacteria, was significantly increased after fermentation of 24 h (BL24h group vs. BL0h group, *p* < 0.05). While for a series of beneficial bacteria such as *Anaerostipes, Collinsella, Blautia*, and *Subdoligranulum*, they were decreased. After fermentation for 24 h, the dominant bacteria for the DO and FDO groups were *Enterococcus, Romboutsia*, and *Bifidobacterium*. They belong to lactic acid bacteria, and could convert carbohydrates to lactic acid or acetic acid, which in turn help to sustain a healthier intestinal microbiota. In addition, the relative abundance of *Clostridium sensu stricto*, a beneficial microorganism in gut microbiota (50), was significantly increased in the FDO6h group (*p* < 0.05). The above results exhibited that DO and FDO could improve the diversity of intestinal flora to some extent, which exerted beneficial regulatory effects.

Taxonomic cladogram produced from LEfSe analysis shows the significant differences in microbiota communities among seven groups, and the enrichment degree of bacterial taxa from phylum to family level (Figure 10C). The LDA effect size analysis identified a total of 135 species-level taxa with significantly differential abundance (Figure 10D). The diversity of the dominant bacteria increased in the FDO group, such as Firmicutes, Actinobacteria, etc.

**Figure 11:**
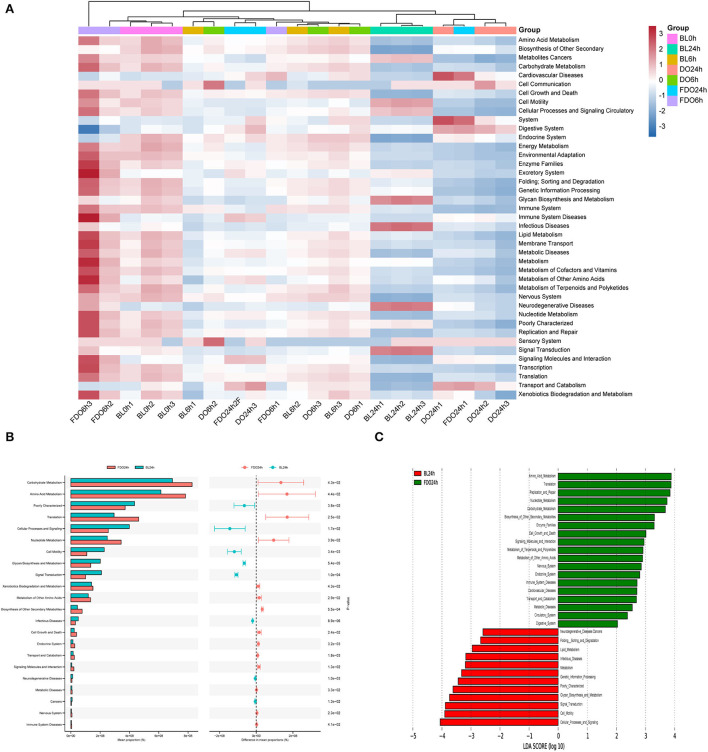
KEGG pathway analysis. **(A)** The heatmap of clustering for KEGG pathways. **(B)** Analysis for KEGG pathway using STAMP. **(C)** Analysis for KEGG pathway using Lefse.

The predicted microbial metabolic functions present against the KEGG database from metagenomic sequences were profiled *via* PICRUSt. The heatmap of clustering for KEGG pathways (Figure 11A) revealed metabolic pathways for all groups at different time points during fermentation. Taken as a whole, most functions were significantly decreased after colonic fermentation (*p* < 0.05). From Figure 11C, the 24 h fermentation in FDO was primarily mediated by the amino acid metabolism, translation, replication and repair pathway. Of those, the carbohydrate metabolism was the most significant difference in the metabolic pathway between FDO and blank at 24 h, which was related to sucrose and propionate (Figure 11B) (51).

## Discussion

As a famous medicinal and edible plant throughout the world, DO is enriched with various biologically active substances, and has great economic value and multiple physiological functions. However, research on the PPs in DO is still scarce. An important factor that limits the functional activity and research use of PPs is its low bioavailability, which strictly depends on its bioaccessibility. Gratifyingly, probiotic fermentation is an effective and green method for improving the bioactivities and bioaccessibility of PPs (52). In addition, the *in vitro* digestion and colonic fermentationmodel also offers a simple, safe, economical, and controllable tool for investigating ways to increase the bioaccessibility of PPs (53).

Our study showed that both *in vitro* digestion and probiotic fermentation could promote the release of phenols and flavonoids in DO. Where the highest PPs levels were found in the intestinal digestion stage among the three stages, which probably was caused by following multiple factors acting singly or in combination: the actions of digestive enzymes, and changes in gastrointestinal pH. Almost during the whole digestion process, FDO processed significantly higher TPC and TFC compared to unfermented samples (*p* < 0.05). Thus, it was indicated that probiotic fermentation also is an effective treatment to improve the bioaccessibility of PPs (54).

Of the ten compounds examined by HPLC analysis, syringic acid, ferulic acid, epigallocatechin gallate, apigenin, and scutellarin were the main five compounds. The number of these compounds gradually decreased from the oral to the intestinal phase but their contents increased in the gastrointestinal phase. Interestingly, p-hydroxybenzoic acid was only identified in FDO during gastrointestinal digestion. While apigenin and scutellarin were major released in colon fermentation, with a maximum level at colon fermentation of 24 h. The above results were attributed to the actions of digestion and fermentation, including formation, transformation, and degradation (55).

To evaluate the effects of probiotic fermentation on the functional activity of DO, the antioxidant activities, α-amylase and α-glucosidase inhibitory activities evaluation were performed. Overall, these bioactivities were found to be strongest in the intestinal phase. When fermented and unfermented groups were compared, FDO showed significantly stronger antioxidant activities and α-amylase inhibitory activity during the digestion process, but there were no obvious rules in the other indexes, that FDO was always stronger or weaker during the whole digestion and colon fermentation process. Considering that probiotic fermentation could result in the increasing and decreasing of the active compound and the production of new compounds in DO (52), and these compounds work together to influence the functional activity of DO, thus, the actions of probiotic fermentation might affect the different bioactivities of DO both in a positive and a negative way. However, in the present study, due to the limiting of experimental conditions, these actions on PPs were not investigated, further relevant in-depth studies such as metabolomics analysis and searching for the key active metabolite were desirable.

Both DO and FDO, especially FDO, could regulate the composition of microbial flora, they could improve the intestinal environment by increasing the populations of lactic acid bacteria, including *Enterococcus, Romboutsia*, and *Bifidobacterium*, and inhibit the growth of pathogenic bacteria. Of those, *Bifidobacterium* could produce short-chain fatty acids to decrease the gut pH and form biological barriers, and secrete anti-microbial compounds to attenuate harmful bacteria (56, 57). Besides, they could increase the output of SCFAs, such as acetic acid and butyric acid, which in turn reduce the intestinal pH value, and improve the intestinal digestive enzyme activities, thus promoting digestion and absorption, and maintaining intestinal environmental stability in colonic fermentation (58). Overall, probiotic fermentation could impact PPs in DO and their metabolites, in turn modulating the colonic microbial composition, and finally improve the digestive stability and bioavailability of PPs in DO.

## Conclusions

Taken together, this study evaluated the effects of probiotic fermentation on the bioactivities and bioaccessibility of PPs in DO under *in vitro* simulated gastrointestinal digestion and colon fermentation, and explored the influence of PPs in DO on fecal microbial diversity. The results showed that probiotic fermentation could increase the release of PPs in digestion, which improved their bioaccessibility. The biological activities of PPs, including antioxidant ability and α-amylase inhibitory activity, were also enhanced after fermentation, and were found to be strongest in the intestinal phase. The PPs in DO could strikingly alter the richness and diversity of fecal microbiota, and probiotic fermentation further promoted this improvement effect. In addition, the PPs both in DO and FDO, especially in FDO, was effective in promoting colonic fermentation, and increasing the concentration of SCFAs. The work revealed potential health benefits of the PPs in DO, and could provide a novel insight into the further utilization of *Dendrobium officinale* by using probiotic fermentation. As the next step, it can be considered to investigate the key polyphenols in *Dendrobium officinale* and their detailed transformation mechanisms during fermentation and digestion further.

## Data availability statement

The datasets presented in this study can be found in online repositories. The names of the repository/repositories and accession number(s) can be found below: BioProject, accession number PRJNA865395.

## Author contributions

RL and ZW: methodology. ZW and KK: validation, writing—review, and editing. PX and XZ: formal analysis. RL: data curation and writing—original draft preparation. PX: supervision. XZ and XH: project administration. XH: funding acquisition. All authors have read and agreed to the published version of the manuscript.

## Funding

This work was supported by Yunnan Agricultural Joint Special General Project (202101BD070001-109 and 202101BD070001-089), China Agriculture Research System (CARS-21), Yunnan Special General Projects of Basic Research (202201AT070049), Yunnan Zhengwenjie Expert Workstation (202205AF150018), Major Science and Technology Project of Yunnan and Kunming (202102AE090042 and 2019ZG0901), Scientific Research Fund Project of Yunnan Provincial Department of Education (2022J0507), and Major Science and Technology Project of Kunming (2021JH002).

## Conflict of interest

The authors declare that the research was conducted in the absence of any commercial or financial relationships that could be construed as a potential conflict of interest.

## Publisher's note

All claims expressed in this article are solely those of the authors and do not necessarily represent those of their affiliated organizations, or those of the publisher, the editors and the reviewers. Any product that may be evaluated in this article, or claim that may be made by its manufacturer, is not guaranteed or endorsed by the publisher.
